# Inclusion of a tannin-rich legume in the diet of beef steers reduces greenhouse gas emissions from their excreta

**DOI:** 10.1038/s41598-022-18523-y

**Published:** 2022-08-20

**Authors:** Flavia O. S. van Cleef, José C. B. Dubeux, Francine M. Ciriaco, Darren D. Henry, Martin Ruiz-Moreno, David M. Jaramillo, Liza Garcia, Erick R. S. Santos, Nicolas DiLorenzo, João M. B. Vendramini, Harley D. Naumann, Lynn E. Sollenberger

**Affiliations:** 1 North Florida Research and Education Center, University of Florida, Marianna, FL 32446 USA; 2 Department of Animal and Dairy Science, University of Georgia, Tifton, GA 31793 USA; 3 U.S. Dairy Forage Research Center, USDA/Agricultural Research Service, Marshfield, WI 54449 USA; 4 Department of Agricultural, Food, and Nutritional Science, University of Alberta, Edmonton, AB T5N3X1 Canada; 5 Range Cattle Research and Education Center, University of Florida, Ona, FL 33865 USA; 6 Division of Plant Sciences, University of Missouri, Columbia, MO 65201 USA; 7 Agronomy Department, University of Florida, Gainesville, FL 32611 USA

**Keywords:** Climate-change mitigation, Environmental impact

## Abstract

The objectives of this study were to determine the emission of nitrous oxide (N_2_O), methane (CH_4_), and carbon dioxide (CO_2_), as well as the isotopic composition of N_2_O from excreta of beef steers fed ‘AU Grazer’ sericea lespedeza hay [SL; *Lespedeza cuneata* (Dum. Cours.) G. Don]. Fifteen Brahman × Angus crossbred steers were fed one of three experimental diets: 0, 50, or 100% inclusion of SL into ‘Tifton 85’ bermudagrass hay (*Cynodon* spp.). Gas sampling occurred on days 0, 1, 3, 5, 7, 14, 18, 25, and 32 after urine or feces application to static chambers for two experimental periods. Effect of the day after feces application (*P* < 0.001), while day × inclusion of SL interaction was observed in urine (*P* < 0.001) for all greenhouse gases (GHG) analyzed. Peaks of emission of all GHG in urine and feces occurred in the first days (*P* < 0.001), with days 3 and 5 being most depleted in ^15^N-N_2_O in feces, and days 3, 5, and 7, in urine (*P* < 0.001). Feeding SL to beef steers was effective in mitigating the emission of GHG from the excreta, but further research is necessary to investigate the mechanisms behind the reductions.

## Introduction

Anthropogenic activity has modified natural environmental processes by increasing atmospheric concentrations of the main greenhouse gases (GHG), including through the expansion of agricultural activities^1^. In 2021, world cattle inventory was reported at 1 billion head^2^, with both enteric fermentation and manure being major contributors of methane (CH_4_) and nitrous oxide (N_2_O)^3^.

Manure is a significant source of GHG in grasslands due to the presence of organic compounds and their decomposition under anaerobic conditions^4^. Anaerobic bacteria decompose the organic material releasing CH_4_, whereas interactions of different sources of N affect the N cycle, thereby, influencing the daily fluxes of N_2_O^5^. The main concern is that the amount of N in the excreta deposited in a specific area exceeds the immediate plant needs, and the excess N is lost through nitrate leaching and N_2_O^6,7^.

Nutritional adjustments are a strategy to modify the intensity and frequency of the processes that lead to the generation and emission of GHG in the excreta of ruminants. Animal diet, as well as the feed quality and quantity can influence urine and feces N concentrations. Forage legumes, for example, provide a wide range of secondary metabolites, such as tannins, that can modulate ruminal fermentation^8^. Condensed tannins (CT) bind to dietary protein protecting it from ruminal degradation, increasing the uptake of amino acids by the small intestine and the excretion of N in the feces^9^. Moreover, while natural nitrogenous compounds in the urine of cattle were reported to inhibit N_2_O formation processes in the soil^10–12^, tannins would affect the proportion of those compounds, which can consequently affect the emissions of N_2_O^13^.

Static chambers are a commonly used technique to measure GHG fluxes from soils, but due to the heterogeneous nature of the fluxes^14^, there are uncertainties in the magnitude, distribution, and temporal pattern of the natural and anthropogenic sources. This limits the accuracy of the results and comparisons among studies from different locations. Within this context, the analyses of the natural abundance of stable isotopes from the gases in the atmosphere can represent a powerful ally to estimate GHG budgets^15^, since the processes of GHG formation and sequestration have specific ratios of heavy to light isotopes^16^.

We hypothesized that the inclusion of a tannin-rich legume in the diets of beef steers would decrease the emission of N_2_O, CH_4_ and CO_2_ from their excreta, due to the shift in N excretion from urine to feces and to the impact of tannins on methanogenesis. Additionally, urine and feces would present different emission factors and isotopic composition of N_2_O, enabling source attribution to the emissions. Therefore, the objectives of this study were to estimate the net emissions of N_2_O, CH_4_, and CO_2_ from excreta of beef steers fed a tannin-rich forage legume, as well as to determine the emission factor of N_2_O and isotopic composition of N_2_O.

## Results

### N_2_O flux and δ^15^N isotopic composition

**Figure 1:**
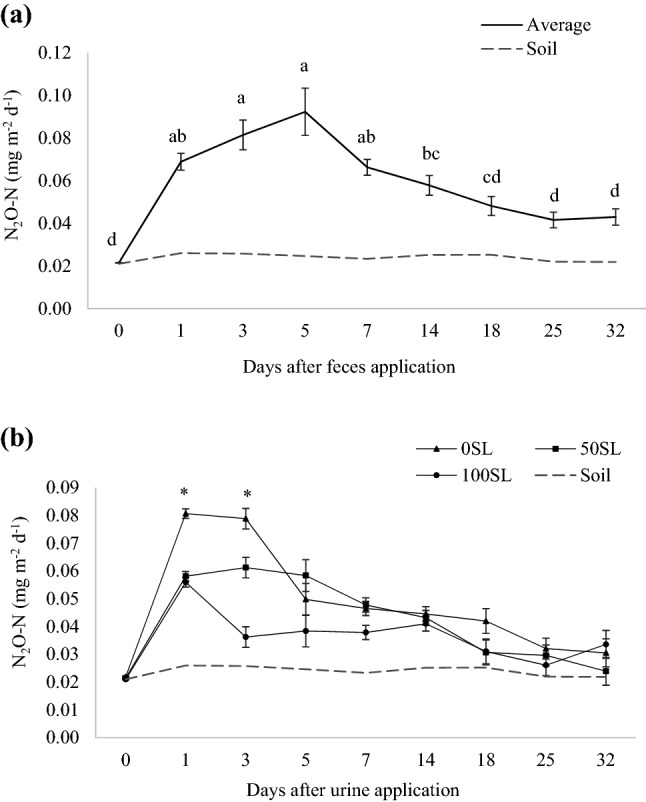
Net flux of N_2_O emitted by (**a**) feces from beef steers fed sericea lespedeza [SL; *Lespedeza cuneata* (Dum. Cours.) G. Don], where values represent the average of three diets (0, 50 or 100% SL) in two experimental periods, and means followed by different letters are significantly different among sampling days (*P*_day_ < 0.001); and (**b**) urine collected from beef steers fed 0SL, 50SL or 100SL, where asterisk indicates differences among diets within day (*P*_SL*Day_ < 0.001). Bars refer to standard deviation. Dashed line represents soil (no excreta) emissions.

There was an effect of sampling day on N_2_O emissions from feces, with the first 5 days presenting greater emissions compared to rest of the days (*P* < 0.001), and intermediate emissions at day 14 (Fig. 1a). For urine, an interaction between SL inclusion level and day of N_2_O emissions was observed (*P* < 0.001), with emissions being significantly greater on days 1, 3, and 5 after urine application (Fig. 1b, P < 0.001), and treatment 100SL presented the least emissions.

Peak N_2_O emissions in feces occurred 5 days after its application for all diets (0.12, 0.07, and 0.08 mg m^−2^ d^−1^ for 0SL, 50SL, and 100SL, respectively) and decreased to similar background emissions after 18 days (0.05 mg m^−2^ d^−1^, *P* > 0.05). On the other hand, peak N_2_O emissions in urine occurred earlier (1 or 3 days after application; 0.08, 0.06, and 0.05 mg m^−2^d^−1^ for 0SL, 50SL, and 100SL, respectively) but took longer (25 days) to reduce to background emissions (0.02 mg m^−2^ d^−1^, *P* > 0.05), despite no significant differences among SL levels being observed from day 7 onward.

**Figure 2:**
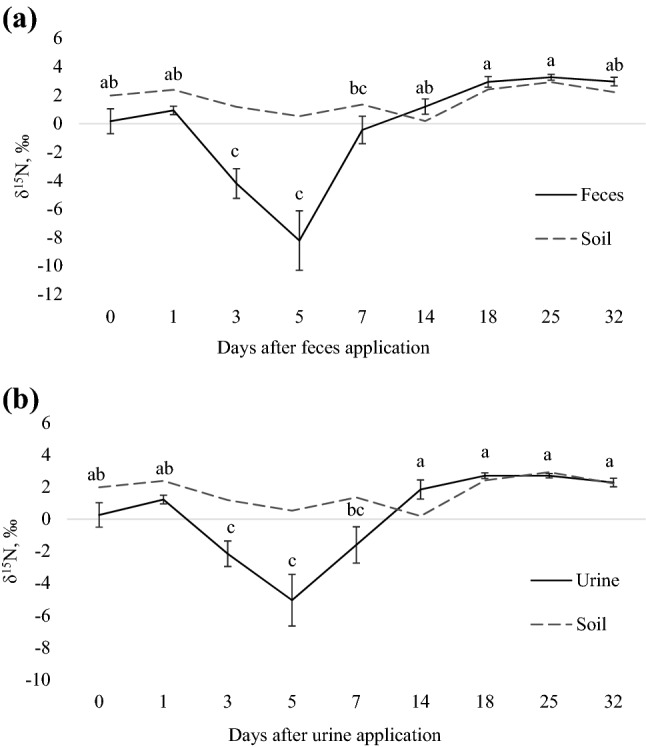
δ^15^N isotopic composition of N_2_O emitted by (**a**) feces from beef steers fed sericea lespedeza [SL; *Lespedeza cuneata* (Dum. Cours.) G. Don], where values represent the average of three diets (0, 50 or 100% SL) in two experimental periods, and means followed by different letters are significantly different among sampling days (*P*_day_ < 0.001); and (**b**) urine patch of beef steers fed 0SL, 50SL or 100SL, where asterisk indicates differences among diets within day (*P*_SL*Day_ < 0.001). Bars refer to standard deviation. Dashed line represents soil (no excreta) emissions.

There were effects of day (*P* < 0.001) on urine and feces δ^15^N composition from N_2_O (Fig. 2). The most δ^15^N depleted N_2_O occurred on days 3 and 5 for both types of excreta (− 4.2 and − 8.2 ‰ for feces, and − 2.2 and − 5.2 ‰ for urine).

### CH_4_ and CO_2_ emissions

**Figure 3:**
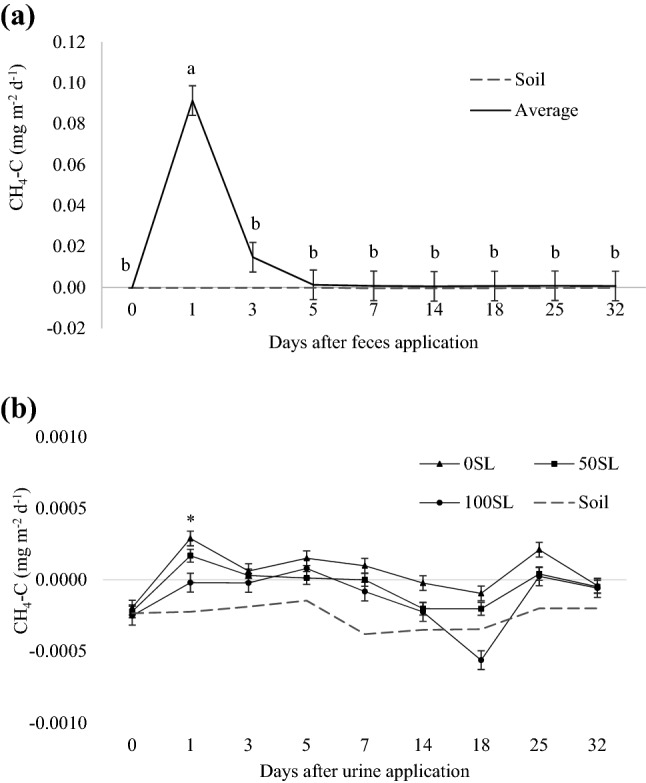
Net flux of CH_4_ emitted by (**a**) feces from beef steers fed sericea lespedeza [SL; *Lespedeza cuneata* (Dum. Cours.) G. Don], where values represent the average of three diets (0, 50 or 100% SL) in two experimental periods, and means followed by different letters are significantly different among sampling days (*P*_day_ < 0.001); and (**b**) urine collected from beef steers fed 0SL, 50SL or 100SL, where asterisk indicates differences among diets within day (*P*_SL*Day_ < 0.001). Bars refer to standard deviation. Dashed line represents soil (no excreta) emissions.

**Figure 4:**
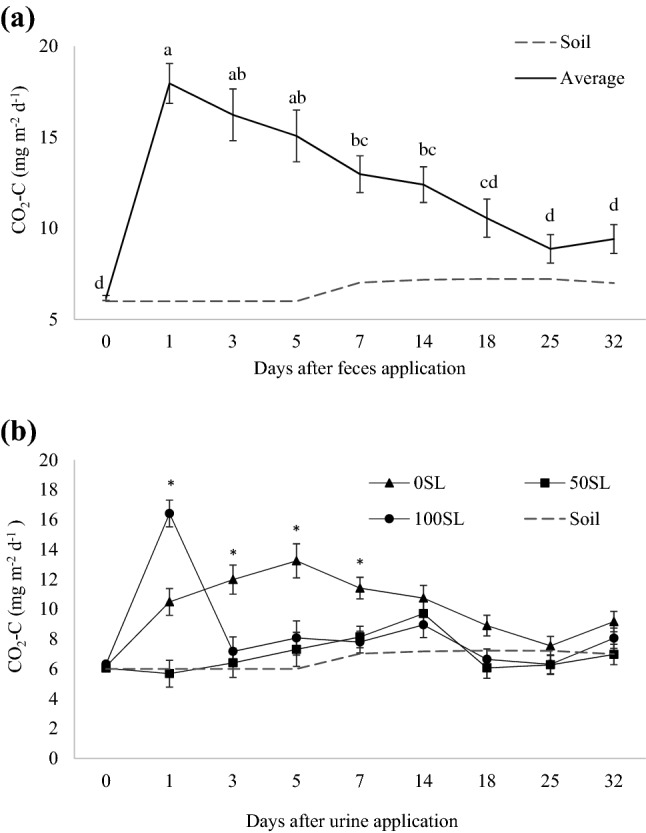
Net flux of CO_2_ emitted by (**a**) feces from beef steers fed 0, 50 or 100% sericea lespedeza [SL; *Lespedeza cuneata* (Dum. Cours.) G. Don], where values represent the average of three diets (0, 50 or 100% SL) in two experimental periods, and means followed by different letters are significantly different among sampling days (*P*_day_ < 0.001); and (**b**) urine collected from beef steers fed 0SL, 50SL or 100SL, where asterisk indicates differences among diets within day (*P*_SL*Day_ < 0.001). Bars refer to standard deviation. Dashed line represents soil (no excreta) emissions.

There were effects of sampling day on CH_4_ and CO_2_ emissions from feces (Figs. 3a and 4a, respectively; *P* < 0.001) and SL inclusion × day interaction on emissions from urine (Figs. 3b and 4b, respectively; *P* < 0.001). For CH_4_, the peak in feces occurred on the first day after the application of excreta, with no significant differences among levels of SL inclusion observed for the other days. In urine, CH_4_ emission was different among the levels of SL only on the first day after its application, with greatest emission demonstrated by treatment 0SL (0.0006 mg m^−2^ d^−1^) and least, by treatment 100SL (− 0.0004 mg m^−2^ d^−1^). Additionally, negative CH_4_ peaks, regardless of the level of SL inclusion, were observed on day 18 after application of urine, which was restored to background levels on day 25.

Peak CO_2_ in feces occurred on day 1 after the application of excreta (*P* < 0.001), regardless of the level of SL, and reductions to background levels were reached at day 18 (Fig. 4b). In urine, although peak was observed on the same day (day 1) for treatment 100SL, peak CO_2_ for treatments 0SL and 50SL occurred 5 or 14 days after excreta application, respectively.

### Excreta composition, cumulative emissions of GHG and N_2_O emission factor

Nitrogen concentration observed in feces was 1.6, 2.2, and 2.8%, while in urine, it was 0.19, 0.24, and 0.58% for 0SL, 50SL and 100SL, respectively (Table 1).

**Table 1:** Dry matter, nitrogen (N), and condensed tannins (CT) concentration of feces and N concentration composition of urine from beef steers fed three levels of inclusion of sericea lespedeza [SL; *Lespedeza cuneata* (Dum. Cours.) G. Don].

Item	Dietary treatments^a^		
	0SL	50SL	100SL
**Feces**			
DM, %	19.1	18.8	19.2
N, %	1.6	2.2	2.8
N, g chamber^−1^	6.1	8.3	10.8
CT, %	ND	2.6	10.7
**Urine**			
N, %	0.19	0.24	0.58
N, g chamber^−1^	3.80	4.90	11.60

*ND* not detected.

^a^0SL: 0% inclusion sericea lespedeza hay (100% bermudagrass); 50SL: 50% inclusion sericea lespedeza hay; 100SL: 100% inclusion sericea lespedeza hay (0% bermudagrass hay).

Cumulative emissions of N_2_O from feces were 2.02, 1.09, and 0.84 mg m^−2^ for 0SL, 50SL, and 100SL, respectively, presenting a negative linear effect of the inclusion of SL (*P* = 0.01; Table 2). In urine, cumulative emissions were 1.44, 0.23 and 0.02 mg m^−2^ for 0SL, 50SL, and 100SL, respectively, also presenting a negative linear effect of the inclusion of SL (*P* = 0.01). The emission factor of N_2_O (EF_N2O_) for each level of inclusion of SL within each type of excreta is described on Table 2.

**Table 2:** Cumulative emissions of greenhouse gases and emission factor of N_2_O-N from feces and urine of beef steers fed three levels of inclusion of sericea lespedeza [SL; *Lespedeza cuneata* (Dum. Cours.) G. Don].

Item	Diets^a^			SEM	*P*-value^b^	
	0SL	50SL	100SL		L	Q
**Cumulative emissions, mg N m**^**−2**^						
Feces						
N_2_O	2.02	1.09	0.84	0.10	0.01	0.05
CH_4_	1.98	0.04	− 0.004	0.06	0.03	0.90
CO_2_	399.72	89.73	15.51	16.4	0.03	0.18
Urine						
N_2_O	1.44	0.23	0.02	0.005	0.01	0.14
CH_4_	0.02	− 0.013	− 0.006	0.008	0.04	0.05
CO_2_	313.74	10.81	8.93	24.9	0.03	0.10
**Emission factor of N**_**2**_**O-N, %**						
Feces	0.09	0.03	0.0002	0.005	< 0.01	0.25
Urine	0.11	0.04	0.009	0.001	< 0.01	0.13

^a^0SL: 0% inclusion sericea lespedeza hay (100% bermudagrass); 50SL: 50% inclusion sericea lespedeza hay; 100SL: 100% inclusion sericea lespedeza hay (0% bermudagrass hay).

^b^BG × SL: bermudagrass versus sericea lespedeza hay inclusion; L: linear effect of SL inclusion; Q: quadratic effect of SL inclusion.

A linear decrease was observed for cumulative emissions of CH_4_ in feces and urine when SL was added (*P* = 0.03 and *P* = 0.04, respectively). In feces, a negative cumulative emission, or an uptake of CH_4_, was demonstrated by the inclusion of 100% SL (− 0.004 mg m^−2^), while in urine, the inclusion of 50% SL presented the least emission (or greatest uptake) in 32 days (− 0.013 mg m^−2^). The cumulative emission of CO_2_ in feces and urine followed a similar pattern of CH_4_, with a linear uptake of CO_2_ for the inclusion of SL (*P* = 0.03) for both excreta types, in which the least emissions were observed for diet 100SL (15.51 and 8.93 mg m^−2^ for feces and urine, respectively).

## Discussion

In a concomitant study, we demonstrated that a great proportion of CT from SL was bound to protein and fiber in the feces of animals fed 100SL, resulting in lesser apparent total tract digestion of those nutrients than animals consuming grass-only diets^17^. Thus, host enzyme digestion and intestinal absorption of amino acids were inhibited by CT binding ability throughout the gastrointestinal tract, which also contributed to greater concentration of N in feces from animals fed 100SL than feces of animals fed 0SL. Moreover, the greater excretion of N in feces may have had a significant contribution from endogenous metabolic N with the addition of CT in the diets^18^, which is consistent with the studies of Komolong et al.^19^ and Carulla et al.^8^.

Greater N concentration in urine of animals fed 100SL than 50SL and 0SL can be also attributable to a high CP intake. High CP intake without a concomitant increase in energy supply overloads ruminal microbes, increasing ammonia production in the rumen, thereby, leading to more ammonia being absorbed by the ruminal wall of animals fed 100SL than 0SL^20^. With that, more ammonia was proportionally being converted to urea and excreted in the urine from animals fed 100SL than those fed the other diets^21^. Broderick^22^ states that feeding ruminants exclusively legumes can result in intense urea production, increasing N excretion in the urine, and thus, reducing N retention by the animals. This is because the rate of ammonia production in the rumen exceeds the rate of use by microorganisms^23^, and if it is not captured as a microbial protein in the rumen, it will undergo the ureagenesis process in the liver.

Beauchemin et al.^24^ supplemented a 70% forage-based diet with 1 and 2% tannins from quebracho (*Schinopsis lorentzii*) and reported a decrease in CP digestibility as well as lesser urinary N, as proportion of total N excretion, when CT was included. They also observed an increase in fecal N excretion, as a proportion of total N excretion, with greater inclusion of CT in the diet, which was attributed to endogenous and microbial contributions, and not an actual decrease in urinary N.

The EF_N2O_ from urine is usually greater than EF_N2O_ from feces^25,26^, since it represents the N in N_2_O as a percentage of the total N applied^27^. Thus, developing an EF for each type of excreta is recommended. In our study, the EF_N2O_ of feces or urine from animals fed SL were lower than feces or urine from animals fed 0SL. Moreover, only EF_N2O_ from feces of animals fed 0SL were similar to the default values provided in the IPCC 2019 refinement^28^, which was based on specific conditions of weather and soil properties and might misrepresent worldwide estimations.

The lack of effect of inclusion of SL in the flux of N_2_O from feces was driven mainly by the presence of organic N in conjunction with the bound-CT^5,17^. The fluxes of N_2_O from urine patches are generally greater than fluxes from feces since urea is more rapidly hydrolyzed than organic N^5^. A review^21^ demonstrated that up to 3.8% of N in urine versus only up to 0.7% of N in feces could be emitted in the form of N_2_O into the atmosphere. However, in the current study, the lesser cumulative emissions from urine patches than from feces pile are probably because urine was being emitted as another compound, such as volatile ammonia.

The fecal pile functions as an organic fertilizer for the soil and nutrient availability is a key component for soil microbiota. According to the “Hole-in-the-pipe” model proposed by Firestone and Davidson^29^, the magnitude of N_2_O production is mainly a function of the availability of N in the soil. Therefore, the availability of mineral N (NH_4_^+^ and NO_3_^−^), as well as the availability of labile carbon, promoted greater N_2_O and CO_2_ emissions in the first days for all treatments, mainly by activation of microbial populations^30^. The deposition of fresh excreta triggered denitrification in the initial days, due to greater moisture and N and other nutrients. Moreover, nitrate leaching losses could also have occurred, which, in part, contributes to indirect N_2_O emissions if nitrate is denitrified within surface waters^31,32^.

According to Franzluebbers and Steiner^33^ temperature, humidity, and soil inorganic N are also determinant of the magnitude of N_2_O emissions. In the current study, rainfall events after application of excreta might have consequently affected the proportion of water-filled pore space (WFPS), creating anaerobic conditions. Hence, the anaerobiosis and the elevated levels of N and C available in the soil due to the deposition of excreta favored nitrification, nitrifier denitrification, and denitrification, resulting in greater GHG production and emissions^34,35^. Posteriorly, with elevated temperatures during summer in Florida, a fast drying of the feces and the formation of a superficial crust were observed^4^, which could also have affected the N_2_O fluxes from feces to decrease from day 5 onward.

Gaseous losses were shown to have a linear relationship with soil δ^15^N, with large fractionation factors if NO_3_^−^ is completely consumed in the denitrification reaction^36^. Reported soil and soil-emitted N_2_O δ^15^N values typically range between 0 and − 40 ‰^37–40^, indicating a wide variation in the depletion of ^15^N in the gaseous loss pathway. In our study, the δ^15^N of N_2_O was more depleted when emissions were more intense (initial days after excreta application), represented by the strong fractionation of ammonia volatilization and the intensification of denitrification process^41^. This trend supports the hypothesis that more N from urine was being lost as ammonia than N_2_O.

Kool et al.^11^ demonstrated that synthetic urine with high hippuric acid concentration reduced N_2_O emissions by up to 50%. More recently, Zhou et al.^13^ attributed the greater reductions in N_2_O emissions from urine to the addition of tannic acid in the diets of beef cattle. Authors concluded that the increased ratio of hippuric acid-N/urinary N had inhibitory effects in the emissions of N_2_O, but the effect of the tannic acid was more evident at greater levels of crude protein. Although we did not determine these compounds in the urine, aromatic compounds from CT present in urine from animals fed 100SL had possibly inhibited the nitrification processes in soil to some extent^42^, resulting in lesser emissions of N_2_O.

Cumulative emissions of all three GHG evaluated were greater for animals fed 0SL than 100SL, regardless of the type of excreta. However, fluxes of CH_4_ were negative after few days the excreta were applied, indicating an uptake from the soil. Moreover, more than 65% of the total emission of all GHG had been emitted by day 7, which demonstrates how emissions are more relevant immediately after excreta deposition^43^.

Methane emissions from excreta occurs mainly because of the decomposition of feces by methanogenic organisms when in favorable anaerobic conditions^44^. Thus, greater fluxes of CH_4_ in the first days after application of excreta, regardless of the type, could be observed, which is similar to those observed in the literature in tropical pastures^25,45^. The methanogenesis was probably favored by the high moisture of the soil in the initial days and tended to decrease with natural decrease of moisture of the excreta, scarcity of rainfall events, and nutrient leaching and consumption by microorganisms^45^. However, CT have been associated with a direct negative effect on methanogens^46^, which might have limited methanogenesis in the excreta of animals fed SL, explaining the linear reduction from 0 to 100% inclusion of the legume in the diets.

In summary, this study showed how diet of the animal, type of excreta, and weather conditions affected GHG fluxes in warm-climate pastures in North Florida. Therefore, feeding beef steers SL was effective in mitigating the emission of N_2_O, CH_4_, and CO_2_ from their excreta for 32 days after deposition in the soil. Furthermore, the EF_N2O_ of the excreta decreased with the inclusion of tannin-rich legume, but feces and urine should be considered separately due to specific variations in each study conditions (diet, soil, precipitation). Thus, the adoption of specific EF_N2O_ for countries and regions would avoid overestimation of global emissions from livestock sector. In the current study, more N was added via excreta of animals fed SL hay, reducing N_2_O emissions compared to excreta of animals fed only grass hay. The δ^15^N followed a similar pattern of the fluxes of N_2_O, which was more depleted at high release rates due to the activity of soil microorganisms after excreta application. Further research is warranted to understand the mechanisms behind the reduced losses, while studies using additional isotope measurements, such as hydrogen isotopes, would facilitate estimates of emission and atmospheric modeling.

## Methods

### Animal care

The study followed all procedures approved by the University of Florida Institutional Animal Care and Use Committee (Protocol #201810218) and all methods were carried out in accordance with relevant guidelines and regulations. This manuscript is reported in accordance with ARRIVE guidelines.

### Experimental site

**Figure 5:**
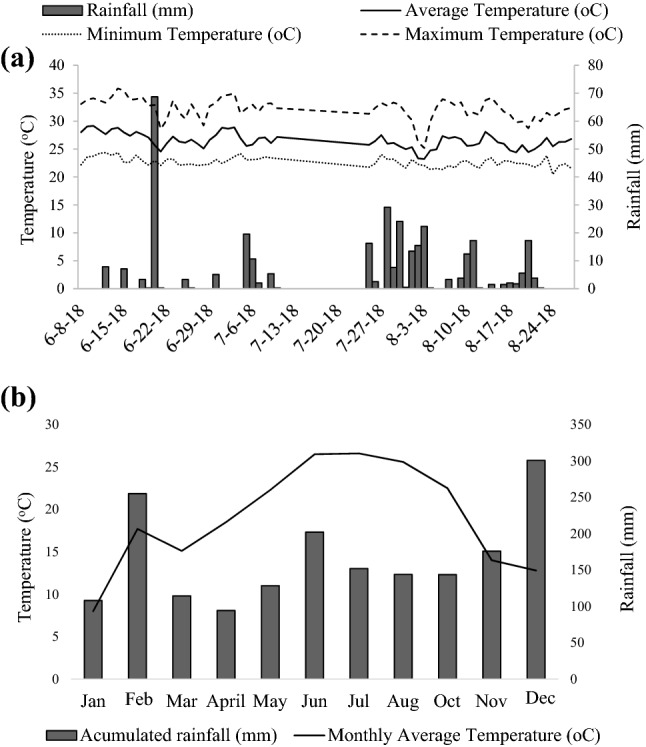
Marianna (FL) station of the Florida Automated Weather Network (FAWN) data of (**a**) weekly rainfall and temperature data from two experimental periods and (**b**) accumulated monthly rainfall (mm) and average temperature (°C). Period 1: from 06/08/2018 to 07/10/2018; Period 2: from 07/25/2018 to 08/27/2018.

The experiment was conducted at North Florida Research and Education Center (NFREC), from University of Florida, located in Marianna, FL (30°52′N, 85°11′ W, 35 m a.s.l.) in a pasture of ‘Pensacola’ bahiagrass (*Paspalum notatum* Flüggé). The soil at the experimental site is classified as an Orangeburg loamy sand (fine-loamy-kaolinitic, thermic Typic Kandiudults), with an average pH of 6.5. Average Mehlich-I extractable P, K, Mg, and Ca concentrations were 13, 45, 31, and 245 mg kg^−1^, respectively. Soil organic matter was 6.3 g kg^−1^, and the estimated cation-exchange capacity was 2.8 meq 100 g^−1^. The study was carried out for two experimental periods of 32 days each, separated by a 15-day interval (Period 1: from 06/08/2018 to 07/10/2018; Period 2: from 07/25/2018 to 08/27/2018). The average, maximum and minimum temperatures and rainfall for the experimental periods are represented in Fig. 5.

### Donor animals and experimental diets

Fifteen Brahman × Angus crossbred steers [Period 1: 324 ± 26 kg initial body weight (BW); Period 2: 336 ± 30 kg BW] were randomly distributed into three experimental diets: 0, 50, or 100% (as fed) inclusion of ‘AU Grazer’ sericea lespedeza hay [SL; *Lespedeza cuneata* (Dum. Cours.) G. Don] into ‘Tifton 85’ bermudagrass hay (BG; *Cynodon* spp.) diets (Table 3) and used as donors of excreta (urine and feces). Steers were fed for 21 days for two feeding periods in a concomitant study^17^ and excreta used in the current study was collected in the last two experimental days of each. All methods were carried out in accordance with relevant guidelines and regulations.

**Table 3:** Chemical composition (DM basis) of experimental diets containing sericea lespedeza [SL; *Lespedeza cuneata* (Dum. Cours.) G. Don] hay fed to beef steers donors of excreta (adapted from van Cleef et al.^17^).

Item	Dietary treatments^a^		
	0SL	50SL	100SL
DM, g/kg	898	922	913
**Nutrient composition, g kg**^**−1**^** DM**			
OM	829	867	857
CP	85	108	161
NDF	747	389	377
ADF	345	323	258
**Condensed tannins, g kg**^**−1**^** DM**			
Bound	ND	23.8	53.5
Unbound	ND	12.5	28.8
Total	ND	36.3	82.3

*ND* not detected.

^a^0SL: 0% inclusion of sericea lespedeza hay (100% bermudagrass); 25SL: 25% inclusion of sericea lespedeza hay; 50SL: 50% inclusion of sericea lespedeza hay; 100SL: 100% inclusion of sericea lespedeza hay (0% bermudagrass hay).

### Static chamber technique

Emissions of GHG were evaluated using the static chamber (non-steady state) technique^14^. Chambers were circular with 30 cm radius and made of a base and a lid, both built out of PVC^47^. The lids were wrapped with reflective tape to provide insulation and a rubber septum was added for gas sampling^48^. The base was fitted with a 10-cm length copper venting tube to ensure adequate air pressure inside the chamber during measurements^49,50^. Lids and bases were kept closed for gas sampling by fitting a bicycle tire inner tube that tightly sealed the parts together.

Bases of chambers were installed in the non-grazed pasture of bahiagrass two weeks prior to excreta application to avoid any effect of soil disturbance on GHG emissions^51^. Bases were installed at 8-cm depth, with 5 cm extending above ground level. Depth for installation was determined based on Clough et al.^48^. Chamber tops were 22 cm height, which when summed with 5 cm of base totaled 27 cm, in agreement with the indication of ≥ 40 cm of chamber height per hour of deployment^48^. New bases were installed in a near location of the same pasture for the second experimental period, also two weeks prior to starting new gas sampling.

### Treatments, excreta deposition, and gas sampling

Treatments applied to the chambers consisted of either feces or urine within one of the three levels of inclusion of SL hay fed to the beef steers and were distributed as a complete randomized block design. Urine and feces were collected directly from each animal by spontaneous or stimulated urinations and defecations and applied at a rate of 2 L of urine and 2 kg of feces, as typical amounts excreted by cattle for the area of the chamber^5,52^. To obtain quantities required of excreta, sampling occurred twice a day (700 and 1500 h) and samples were kept refrigerated at 4 °C until next morning (day 0 of gas sampling). Samples of each excreta type were composited across all five animals within each SL diet resulting in three final subsamples (urine and feces from each of three SL diets). Excreta samples were kept at room temperature 2 h prior to application to the chambers and their chemical composition is described on Table 1.

Application of excreta to the chambers was made one time in each experimental period on the soil surface inside the area determined by the base of the chamber (0.28 m^2^^53^). Grass inside the chamber area was cut at ground level before each sampling day, when appropriate. Gas sampling occurred between 0900 and 1100 h, when temperature is considered more representative of the daily average^47^ on days 0, 1, 3, 5, 7, 14, 18, 25, and 32 after excreta application for both experimental periods. One subsample was taken per deployment time per chamber, separated by 15-min intervals (T_0_, T_15_, and T_30_). At T_0_, a sample was collected from the area directly above the soil surface^54^. Immediately thereafter, chambers were tightly closed by fitting the lid to the base with the bicycle inner tube, followed by the next sample deployment times. All samples were collected with the use of a 60-mL syringe and immediately flushed into a pre-vacuumed 30-mL glass vial. The vial was equipped with a butyl rubber stopper and sealed with an aluminum septum. Samples were analyzed immediately after finishing each experimental period.

Gas sample analyses were conducted using a gas chromatograph (Trace 1310 Gas Chromatograph, Thermo Scientific, Waltham, MA). For N_2_O, an electron capture detector (350 °C) and a capillary column (J&W GC packed column in stainless steel tubing, length 6.56 ft (2 M), 1/8 in. OD, 2 mm ID, Hayesep D packing, mesh size 80/100, pre-conditioned, Agilent Technologies) were used. Methane was analyzed using a flame ionization detector (250 °C) and a capillary column (J&W PoraBOND Q GC Column, Agilent Technologies). For CO_2_, a thermal conductivity detector (200 °C) and capillary column [J&W GC packed column in stainless steel tubing, length 7 ft (2.13 M), 1/8 in. OD, 2 mm ID, Haysep N packing, mesh size 60/80, pre-conditioned, Agilent Technologies] were used. Temperature of the injector and columns were 80 and 200 °C, respectively.

### Calculations and δ^15^N-N_2_O composition

The hourly gases fluxes (mg of N_2_O or CH_4_ or CO_2_ per m^−2^ h^−1^) were calculated according to Cardoso et al.^55^:

$${\text{F}}_{{{\text{GHG}}}} = \left( {\updelta {\text{C }}/ \updelta {\text{t}}} \right) \, \times \, \left( {{\text{V}}/{\text{A}}} \right) \, \times \, \left( {{\text{M}}/{\text{Vm}}} \right),$$

where δC/δt is the change in gas concentration in the chamber during the deployment time; V and A are the chamber volume and soil area covered by the chamber, respectively; M is the molecular weight of the gas; and Vm is the molecular volume of gas. The Vm parameter was corrected to the standard conditions of temperature and pressure as Vm = 0.02241 × (273.15 + Tc/273.15) × p0/p1, where 0.02241 is the molar volume (m^3^), Tc is the chamber headspace temperature at sampling time (°C), p0 is the air pressure at sea level, and p1 is the local pressure calculated using the barometric equation. The minimal detectable flux was 0.012 ppb min^−1^ for N_2_O, 0.004 ppm min^−1^ for CH_4_, and 1.40 ppm min^−1^ for CO_2_.

Daily N_2_O, CO_2_, and CH_4_ emissions were calculated by multiplying the fluxes by 24 h and cumulative emissions were estimated by integrating the fluxes over each day (area under the curve) and averaged per period. The fraction of N applied in the excreta lost as N_2_O, named as emission factor (EF), was calculated according to the equation:

$${\text{EF}}_{{{\text{N}}_{{2}} {\text{O}}}} \left( \% \right) \, = \, \left[ {\left( {{\text{N}}_{{2}} {\text{O}} - {\text{N}}_{{{\text{emitted}}}} } \right) \, - \, \left( {{\text{N}}_{{2}} {\text{O}} - {\text{N}}_{{{\text{blank}}}} } \right)} \right]/{\text{N}}_{{{\text{applied}}}} \times { 1}00,$$

where ${\text{EF}}_{{{\text{N}}_{{2}} {\text{O}}}}$ is the emission factor of N_2_O; N_2_O-N_emitted_ is the cumulative N_2_O-N emissions from the chamber with excreta (mg m^−2^); N_2_O-N_blank_ is the cumulative N_2_O-N emissions from the blanks (chamber with no excreta deposited; mg m^−2^); and N_applied_ is the urine or feces N application rate (mg m^−2^).

A subsample (12 ml) of the collected gas was transferred to evacuated exetainers (Labco, UK). Exetainers were pierced with a double needle and flushed with an ultrapure stream of He (12 mL min^−1^) for 6 min using an autosampler (Gilson GX-271, Gilson Inc, WI). During flushing, samples were transferred to a preconcentration unit (Trace Gas, Hanau, Germany) equipped with glass traps (OD 10 mm, length 20 cm) filled with Ascarite, Sofnocat and Mg(ClO_4_)_2_ to scavenge CO_2_, CO, and water, respectively. Remaining N_2_O was cryo-focused on a capillary column submerged in liquid nitrogen for 12 min and transferred to an isotope ratio mass spectrometer (IsoPrime 100, IsoPrime, Manchester, UK) for ^15^N analysis, using He (2 ml min^−1^) as a carrier. The isotope ratio for ^15^N/^14^N was calculated as:

$$\updelta^{{{15}}} {\text{N }} = \, \left( {^{{{15}}} {\text{N}}/^{{{14}}} {\text{N}}_{{{\text{sample}}}} -^{{{15}}} {\text{N}}/^{{{14}}} {\text{N}}_{{{\text{reference}}}} } \right)/\left( {^{{{15}}} {\text{N}}/^{{{14}}} {\text{N}}_{{{\text{reference}}}} \times { 1}000} \right),$$

where δ^15^N is the N isotope ratio of the sample relative to atmospheric nitrogen, ^15^N/^14^N_sample_ is the N isotope ratio of the sample, and ^15^N/^14^N_reference_ is the N isotope ratio of atmospheric N (standard). The stable isotopic composition of nitrogen was reported using the conventional delta per mill notation. δ^15^N values are expressed relative to the international standard (AIR-N_2_).

### Statistical analyses

The experiment was analyzed as a completely randomized block design, with feces and urine data computed separately due to differences in the magnitude of responses^53^. There were three replicates (chamber) of each treatment, and day was considered the repeated measurement for all variables. Glimmix procedure of SAS (SAS Inst., Inc., Cary, NC, version 9.4) was used, in which the chamber was considered the experimental unit. Graphs were drawn using Microsoft Excel (version 16.61). Normality of distribution and homogeneity of variances were evaluated using the Univariate procedure of SAS. Covariance structures were based upon the smallest Akaike Information Criterion value. The model included the fixed effect of level of SL inclusion and day after excreta application and their interaction, and the random effects of block, period, and their interactions. Means were compared using the PDIFF adjusted by Tukey’s test at 5% significance.

## Data Availability

All data generated or analyzed during this study are included in the article.
